# Long-term trihexyphenidyl exposure alters neuroimmune response and inflammation in aging rat: relevance to age and Alzheimer’s disease

**DOI:** 10.1186/s12974-016-0640-5

**Published:** 2016-07-01

**Authors:** Yuqi Huang, Zhe Zhao, Xiaoli Wei, Yong Zheng, Jianqiang Yu, Jianquan Zheng, Liyun Wang

**Affiliations:** State Key Laboratory of Toxicology and Medical Countermeasures, Beijing Institute of Pharmacology and Toxicology, Beijing, 100850 Peoples’ Republic of China; Department of Pharmacology, Ningxia Medical University, 1160 Shengli Street, Yinchuan, 750004 Ningxia Peoples’ Republic of China

**Keywords:** Neuroimmune response, Anticholinergic drug, Neurodegeneration, MHC class I, Microglia activation, Cognitive impairment

## Abstract

**Background:**

Clinical studies have shown an association between long-term anticholinergic (AC) drug exposure and Alzheimer’s disease (AD) pathogenesis, which has been primarily investigated in Parkinson’s disease (PD). However, long-term AC exposure as a risk factor for developing neurodegenerative disorders and the exact mechanisms and potential for disease progression remain unclear. Here, we have addressed the issue using trihexyphenidyl (THP), a commonly used AC drug in PD patients, to determine if THP can accelerate AD-like neurodegenerative progression and study potential mechanisms involved.

**Methods:**

Male Sprague-Dawley rats (SD) were intraperitoneally injected with THP (0.3 and 1.0 mg/kg) or normal saline (NS) for 7 months. Alterations in cognitive and behavioral performance were assessed using the Morris water maze (MWM) and open field tests. After behavior tests, whole genome oligo microarrays, quantitative real-time reverse transcription-polymerase chain reaction (qRT-PCR), immunohistochemistry, and immunofluorescence-confocal were used to investigate the global mechanisms underlying THP-induced neuropathology with aging.

**Results:**

Compared with NS controls, the MWM test results showed that THP-treated rats exhibited significantly extended mean latencies during the initial 3 months of testing; however, this behavioral deficit was restored between the fourth and sixth month of MWM testing. The same tendencies were confirmed by MWM probe and open field tests. Gene microarray analysis identified 68 (47 %) upregulated and 176 (53 %) downregulated genes in the “THP-aging” vs. “NS-aging” group. The most significant populations of genes downregulated by THP were the immune response-, antigen processing and presentation-, and major histocompatibility complex (MHC)-related genes, as validated by qRT-PCR. The decreased expression of MHC class I in THP-treated aging brains was confirmed by confocal analysis. Notably, long-term THP treatment primed hippocampal and cortical microglia to undergo an inflammatory phenotypic switch, causing microgliosis and microglia activation, which were positively accompanied by pathological misfolded tau lesions.

**Conclusions:**

Our findings suggest that immune response and neuroinflammation represent a pivotal mechanism in THP-induced AD-like neuropathology processes with long-term exposure to AC drugs.

**Electronic supplementary material:**

The online version of this article (doi:10.1186/s12974-016-0640-5) contains supplementary material, which is available to authorized users.

## Background

Negative cognitive effects are known to be an adverse effect of anticholinergic (AC) drugs and are assumed to be transient and reversible [1–3]. However, there is a new emerging hypothesis for the connection between AC exposure and pathogenesis of Alzheimer’s disease (AD), with the primary clinical lead for this connection being Parkinson’s disease (PD) [4, 5]. Trihexyphenidyl (THP) is the most commonly used AC drug in PD patients. Perry et al. found that continuous THP use for at least 2 years doubled the prevalence of both amyloid plaque and neurofibrillary tangle densities in PD patients [4–6]. Furthermore, some animal studies demonstrated that use of ACs increased Aβ peptide presence in the cortex and hippocampus, while selective M1 agonists are efficacious for AD treatment [7, 8]. Therefore, duration of AC drug administration has been described as a risk factor for appearance of dementia in PD patients. Despite early epidemiological evidence supporting this hypothesis in the clinic, understanding of the impact of ACs on pathogenesis of neurodegeneration is currently limited. In particular, it is not clear whether the onset of events that results in AC administration is part of aging or reflects early AD development. Understanding such processes is vital as most drug therapies involve chronic administration.

In the current study, we examined the hypothesis that duration of AC drug exposure affects progression of central nervous system (CNS) neurodegeneration. We treated rats with THP (0.3 or 1.0 mg/kg/day, intraperitoneal (IP)) for 7 months; these concentrations have proven successful in previous studies [9, 10]. Administration was started in 3-month-old normal Sprague-Dawley (SD) rats, while the control group was provided with normal saline (NS) only. Behavioral performance was assessed using the Morris water maze (MWM) and open field tests. Additionally, after behavioral testing, whole genome oligo microarrays and quantitative real-time reverse transcription-polymerase chain reaction (qRT-PCR) were performed in the hippocampus to gain insight into the global gene expression and pathway responses to long-term THP exposure. Further, the cerebral cortex and hippocampus were histologically examined for associated neurodegeneration. We found that THP treatment altered neuroimmune responses and promoted CNS neuroinflammation features consistent with microgliosis and microglia activation. We believe that such detailed information is critical for understanding the long-term effects of ACs on occurrence or earlier development of AD.

## Methods

### Chemicals

Trihexyphenidyl (THP) hydrochloride was purchased from Sigma (St. Louis, MO, USA). All drugs were dissolved or diluted in NS, and solutions adjusted to a pH range of 6.5–7.0 for IP injections.

### Animals and experimental design

Adult male SD rats (180–200 g) were supplied by the Animal Center of the Academy of Military Medical Science (Beijing, China). All rats were kept in plastic cages and received food and water ad libitum. The colony room was maintained at 22–23 °C and kept on a 12-h light/dark cycle. All testing was performed under the guidelines for the use of experimental animals of the Beijing Local Committee on Animal Care and Use.

### Long-term THP treatment

**Fig. 1:**
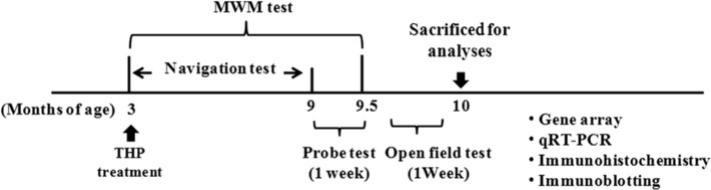
Study timeline. Study consisted of long-term THP treatment, in which male SD rats received THP (0.3 or 1.0 mg/kg/day, IP) or control solution (NS) for an average period of 7 months, beginning at 3 months of age. Rats first underwent the MWM navigation test during the 6-month THP treatment. After the last navigational training test, probe test were performed three times within 1 week. After a 1-week interval, the open field test was then performed. After completion of behavioral testing, the rats were sacrificed for microarray, immunohistochemistry, immunoblotting, and quantitative real-time reverse transcription-polymerase chain reaction (qRT-PCR) analyses

Long-term THP treatment was initiated at 3 months of age (*n* = 10 per group). Each rat was given a single IP injection of THP (0.3 or 1.0 mg/kg/day) in a volume of 0.1 ml/100 g body weight. Controls received an equivalent volume of NS. Treatment was maintained during behavioral testing. Figure 1 schematically depicts the experimental timeline. After injection, animals were immediately placed back in their home cage and left undisturbed.

### Behavioral testing

#### Water maze test

A MWM apparatus (Chengdu Technology & Market Co. Ltd., Chengdu, China) was used to test spatial learning and memory, as previously described [11], with minor modifications. Briefly, the animals received three trials per day for 7 days to learn the task. Each trial lasted until the animal found the platform or for a maximum of 120 s. Animals that failed to find the platform within 120 s were guided there by the experimenter.

After the learning trail, rats were assigned to THP-treated (0.3 or 1.0 mg/kg; *n* = 10 per group) or NS-treated (*n* = 10) groups based on the same average escape time to reach the platform. For subsequent navigation tests, the animals underwent six consecutive training sessions for 6 months. For each training session, the animals received three trials per day for 7 days at the beginning of each month. On each trial, rats were placed into the pool facing the wall, with start locations varied across trials and days. The time taken to reach the platform was recorded as the escape latency(s). During the navigation test, escape latency to the platform for all trials was recorded. Spatial learning ability was determined by the average escape latency to the platform in every session. Swimming speed was averaged according to the last trial.

The probe trial was performed three times within 1 week following the final day of navigation training. On the day of testing, animals were given a probe trial, with the platform removed. All trials were recorded for 60 s using a digital camera. Additionally, the time spent in each target quadrant, as well as the number of annulus crossings (defined as the circular zone surrounding the platform position) was recorded to evaluate spatial memory ability. All training took place during the light phase, between 8:00 AM and 12:00 PM (lights on at 6:00 AM).

#### Open field activity

After completion of the MWM test, the animals were given 1 week of rest, and then spontaneous activity was assessed using a Digiscan Animal Activity Monitor system for 5 min (Jiliang Software Technology Co., Ltd., Shanghai, China), as described previously [12]. Each activity unit contains 16-photo beams positioned 5 cm apart: 8 on the *X*-axis and 8 on the *Y*-axis. The Digiscan analyzer was interfaced with an IBM XT computer using JLBehv software (Jiliang Software Technology Co., Ltd.). On the day of testing, the rats were transferred from the animal colony into the laboratory and allowed to habituate for 120 min before testing. Total movement activity was recorded for each experiment. Inner or outer time measures the proportion of time spent in the center or outer of the arena, respectively (i.e., percentage of total time).

#### RNA extraction and microarray analysis

All samples were hybridized onto the Whole Rat Genome Oligo Microarray (Agilent Technologies, Madrid, Spain) encompassing more than 44,000 rat DNA probes. Protocols for the sample preparation and hybridization were adapted from the Agilent Technical Manual. RNA extraction and microarray analysis were performed as described previously [13]. RNA was isolated using the Qiagen RNeasy Mini Kit for animal tissues (Qiagen, Inc., Valencia, CA, USA). RNA quality and quantity was checked using the Agilent 2100 bio-analyzer and by running RNA sample aliquots on the Affymetrix Gene Chip (Agilent Technologies). Total RNA was used to generate biotin-labeled cRNA using the MessageAmp™ Premier RNA Amplification Kit (cat #1972; Ambion, Austin, TX, USA). A total of 0.75 μg of biotin-labeled cRNA was hybridized onto chips by incubating at 65 °C for 17 h with constant rotation using the Eukaryotic Hybridization Control Kit (Affymetrix, San Diego, CA, USA). Arrays were washed, blocked, and labeled cRNA detected by staining with Fluidics Station 450/250 Cocktail (Affymetrix). Arrays were scanned using an Affymetrix GeneChip® Scanner 3000, and image data extracted using AGCC Software (Affymetrix GeneChip Command Console® Software). All results were given as gene expression ratio (ratio of Cy3 to Cy5 intensity). Functional annotations (including Gene Ontology (GO) and Kyoto Encyclopedia of Genes and Genomes (KEGG) pathways) were analyzed using the Molecule Annotation System (MAS) functional tool (http://bioinfo.capitalbio.com/mas3/). To detect THP-responsive genes, biological pathways that were likely to be involved in THP action in the nervous system were identified using typical criteria (fold change >2). These genes were then assigned to relevant GO biological pathways and KEGG molecular pathways by MAS. Related, significant GO biological pathways were identified by EASE score *P* values <0.01, while significant KEGG molecular pathways were identified using EASE score *P* values <0.05. Accordingly, higher enrichment and gene counts deemed that the genes of these pathways were more important.

#### Quantitative real-time reverse transcription-polymerase chain reaction

To verify the cDNA microarray results, qRT-PCR was performed for six selected genes: MHC class I RT1.Ac heavy chain, signal peptide (*RT1-CE12*); histocompatibility 2, class II antigen E alpha (*R-H2Ea*), class II invariant chain (*Cd74*), major histocompatibility complex, class I-related (*Mr1*); and collagen, type I, alpha 2 (*COL1a2*), and MHC class I RT1 class Ib, locus Aw2 (*RT1-AW2*). Real-time PCR was performed with the primers listed in Table 1 as described previously [14], qRT-PCR was performed using the Light Cycler FastStart DNA Master SYBR Green I kit (Roche Diagnostics, Mannheim, Germany), according to the manufacturer’s instructions, on a Roche Light Cycler platform (Roche, Göttingen, Germany). cDNA was used for each analyzed sample. A calibration curve was included in each experiment (four serial dilutions). Final products were analyzed using the provided software (Roche Molecular Biochemicals LightCycler Software v3.5). Melting curves occurred at 95 °C for 60 s and 55 °C for 30 s. All quantifications were normalized to the β-actin gene. The six genes (*RT1-EC12*, *R-H2Ea*, *Mr1*, *Cd74*, *Colla2*, and *RT1-AW2*) were chosen for qRT-PCR validation according to the criteria of (1) high within-group homogeneity of microarray values and high fold change (FC > |2|) or (2) known major histocompatibility complex (MHC)-relative immune function.

**Table 1:** Sequence of specific primers used for quantitative real-time PCR (qRt-PCR)

Gene ID	Primer	Pair sequences (5′-3′)	Length (bp)
NM_031144	R-β-actin-S	TGCTATGTTGCCCTAGACTTCG	240
	R-β-actin-A	GTTGGCATAGAGGTCTTTACGG	
NM_012645.1	R-RT1-AW2-S	TGGGCTTCTACCCTGCTGACA	138
	R-RT1-AW2-A	CAAGAGGCACCACCACAGATG	
NM_001008847.2	R-H2Ea-S	TCCACTATCTGACCTTCCTGCC	129
	R-H2Ea-A	CTTTAGTTTCTGGGAGGAGGGTT	
NM_001100635.1	R-MR1-S	CGGGTGACGCTGTCGTATGTAG	172
	R-MR1-A	TGTTCCTGCTACCGTTCCTCAC	
NM_001008835	R-RT1-CE12-S	TGAACCTGAGGACACTGCTTGG	218
	R-RT1-CE12-A	CTCCCACTTGTTTCGGGTCATC	
NM_053356.1	R-Col1a2-S	GTGCCTAGCAACATGCCAATCT	207
	R-Col1a2-A	TGAGCAGCAAAGTTCCCAGTAAG	
NM_013069.2	R-CD74-S	TCTGGACTGGAAGGTCTTTGAG	162
	R-CD74-A	CCCATATCCTGCTTGGTCACT	

A non-template control with non-genetic material was included to eliminate contamination or nonspecific reactions. Each sample (*n* = 4) was tested in triplicates and then used for the analysis of relative transcription data using the 2^−ΔΔCT^ method (Table 1).

#### Immunohistochemistry and immunofluorescence

Paraffin wax-embedded rat brain sections (4 μm thick) were dewaxed in xylene (three times for 3 min) and rehydrated in decreasing concentrations of ethanol (100 % twice, 96 %, 70 % ethanol, and H_2_O twice for 3 min each), followed by washing for 5 min in 50 mM Tris-buffered saline (TBT) (pH 7.4). Optimal antigen retrieval was achieved by treating sections for 5 min with a citrate buffer (0.1 M citric acid and 0.1 M tri-sodium citrate-2-hydrate [15]) and microwaving at 84 °C followed by pepsin treatment, as described. Before primary antibody incubation, sections were treated for 1 h with blocking solution (phosphate-buffered saline (PBS) (pH 7.4) containing 5 % normal horse serum, 5 % normal goat serum, and 4 % BSA). Primary antibodies were diluted in PBS (pH 7.4; with 2.5 % normal horse serum, 2.5 % normal goat serum, and 2 % BSA).

Cortical or hippocampal sections from rats were incubated overnight at 4 °C in a moist chamber with rabbit CD11b (1:100), goat anti-Iba1 (1:100), rabbit p-tau AT270 (p-T181) (1:300), and phospho-tau AT8 (p-S202/T205) (1:300) in TBS-T with 1 % BSA. After a further round of washing with TBS-T, sections were incubated with DAB-conjugated secondary antibodies (1:250; Jackson ImmunoResearch, Bar Harbor, ME, USA) for 1 h in a moist chamber at room temperature. All antibodies were purchased from Abcam (Shanghai, China). Slides were then rinsed in TBS-T, mounted in a mix of glycerol/PBS (3:1), and observed using an inverted microscope (Nikon eclipse TS100, Nikon, Tokyo, Japan) equipped with a digital camera (DXM1200F).

For immunofluorescence staining, cortical or hippocampal sections were collected consecutively, and then incubated overnight at 4 °C with anti-rat MHC class I (MHCI) (ER-HR52) (1:100), mouse anti-NeuN (1:200), rabbit anti-M1 receptor (1:100), and mouse CD68 (1:100) (Abcam). After washing with PBS, sections were incubated with FITC-Green 488- or TRITC-Red 595-conjugated secondary antibody (1:500) for 2 h at 37 °C. Images were observed by laser confocal microscope (LSM710; Zeiss, Germany). Cropping of images and adjustment of brightness and contrast were identical for each staining and performed using Adobe Photoshop CS5 software (Adobe Systems, San Jose, CA, USA).

#### Immunoblotting

Equal protein amounts were separated on 4–12 % SDS-PAGE gradient gels, transferred to nitrocellulose membranes, and incubated overnight with primary antibody at 4 °C. The following primary antibodies were used: phospho-tau AT8 (p-S202/T205) (1:1000), p-tau AT270 (p-T181) (1:1000), CD11b (1:1000), and CD68 (1:1000). After washing, membranes were incubated with secondary antibody coupled to horseradish peroxidase. Immunocomplexes were visualized using the Super Signal West Pico Kit from GE Healthcare (Amersham). Band density measurements were obtained using ImageJ 1.36b imaging software (National Institutes of Health, Bethesda, MD, USA).

#### Gallyas silver staining

To identify mature neurofibrillary pathology in neurons, *Gallyas* silver iodide staining was performed [16]. Sections were examined using an Olympus BX51 microscope and photographed using an Olympus camera DP-50 (Olympus, Japan).

#### Fluoro-Jade B staining

A commercial Fluoro-Jade B (FJB) kit (Millipore, Billerica, MA, USA) was used to detect dystrophic neuritis. Tissue sections processed for anti-NeuN immunofluorescence were mounted on gelatin-coated slides, air dried for 2–3 h, and then FJB stained in accordance with the manufacturer’s protocol. In brief, slides were incubated in a 0.06 % potassium permanganate solution for 10 min, followed by a 0.0004 % FJB solution for 30 min. Sections were washed three times in distilled water, fully dried, and cleared in xylene. Slides were coverslipped using aqueous mounting medium containing DAPI (Dako, Glostrup, Denmark) and examined under a confocal laser scanning microscope. After immersion in xylene, integrated immunofluorescence density analysis was performed using ImageJ software (National Institutes of Health, Bethesda, MD, USA). The integrated pixel density was measured in three random areas from each slide (four slides in total). The average integrated pixel density was calculated for each slide and each group, and then compared.

### Statistical analysis

All data were expressed as mean ± S.D. Student’s *t* tests were used to determine differences between the two experimental groups, while one-way or two-way analysis of variance (ANOVA) was used to compare differences between three or more groups for individual group comparisons. Data were analyzed using GraphPad Prism 5.0 software (GraphPad Software Inc., San Diego, CA). The accepted level of significance for all tests was *P* < 0.05.

## Results

### Cognitive behavioral impairment is present during early THP treatment.

**Fig. 2:**
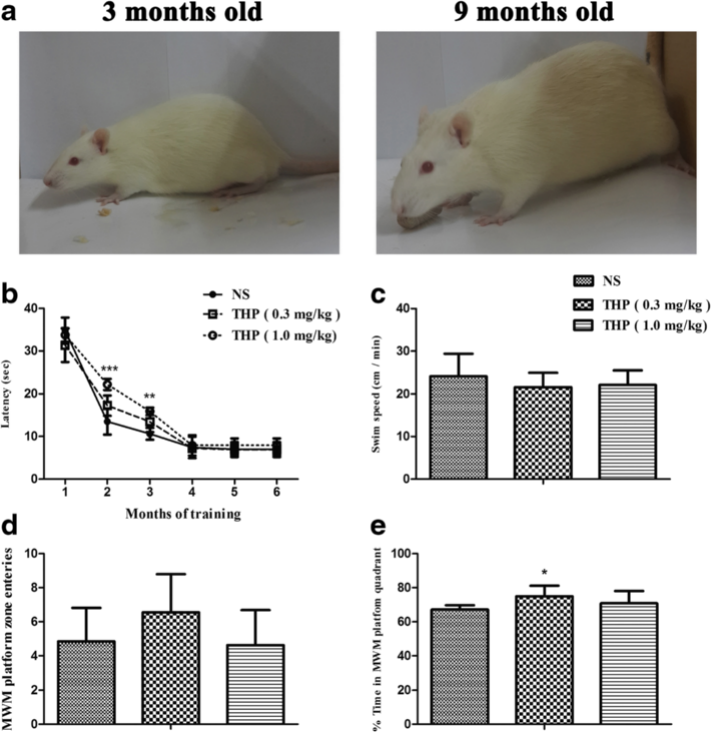
Effect of THP on learning and memory in SD rats after 6 months of treatment. **a** Three- and 9-month-old rats were tested in the MWM. **b** THP/NS effects on MWM platform acquisition measured by escape latency (in seconds). The effect on platform escape latency was measured for 7 days at the beginning of every month over 6 months of MWM testing. **c** THP/NS effect on swimming speed when searching for the MWM platform. **d** THP/NS effect on number of platform crosses during the three-probe trial time period after MWM acquisition. **e** THP/NS effect on time spent in the MWM platform quadrant during the three-probe trial time period after MWM acquisition. The THP (0.5 mg/kg) group spent significantly more time in the MWM platform quadrant. For this and subsequent analyses, histogram values represent mean ± SD (*n* = 10). *P < 0.05, **P < 0.01, ***P < 0.001

The effect of long-term THP treatment on cognitive capacity of the aging rat was assessed using the MWM test. THP (0.3 and 1.0 mg/kg/day) was administered intraperitoneally for 6 months to determine its long-term effect on memory deficits. The MWM test was initiated at 3 months of age. Age-matched, NS-treated littermates were maintained as the control group. Rats at 3 and 9 months of age were treated as younger and aging rats, respectively, in our study (Fig. 2a). Their performance in the MWM task is shown (Fig. 2). As shown in Fig. 2b, NS-treated littermates showed a typical reduction in escape latency time at the beginning of 3 months of training (Fig. 2b), while THP-treated rats showed cognitive impairment, as indicated by longer latencies to find the platform. Accordingly, THP (1.0 mg/kg/day) significantly extended the time to find the escape platform at the 2-month (*t* [10] = 9.226, *P* < 0.001) and 3-month (*t* [10] = 5.539, *P* < 0.001) training sessions. However, upon repetition of training in the subsequent trials at 4 to 6 months, THP-treated rats showed a progressive reduction in the time to reach the escape platform, with the escape latency reduced to the same level as NS control rats (approximately 7.02 ± 1.44 s) (Fig. 2b). At the end of navigation training, swimming speed was not significantly different between groups (Fig. 2b). After removal of the hidden platform, a 60-s probe trial was performed to assess the animal’s memory for the platform location. THP (0.3 mg/kg) slightly increased the number of platform crossings, and one-way repeated measures ANOVA revealed a significant group effect (*F* [1, 10] = 4.6, *P* < 0.05; Fig. 2d). The time spent in the relevant quadrant zone during the probe trial for NS-treated rats was 67.22 ± 2.5 %, whereas for THP-treated rats was slightly more specific (70.88 ± 7.26 % for 1.0 mg/kg and 74.98 ± 6.27 % for 0.3 mg/kg; Fig. 2e). These findings indicate that THP-treated rats effectively retained memory of the platform position at the end of the MWM test.

**Fig. 3:**
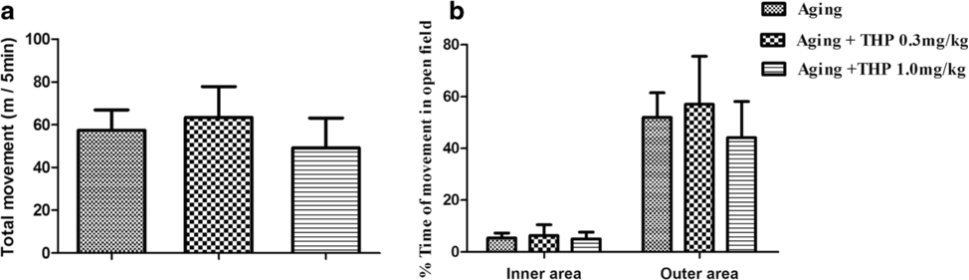
THP/NS effect on open field test performance. **a** Total count for movement activity. **b** Travelled time spent in specific zones of the apparatus. The THP group displayed similar behavior with no difference between groups in terms of movement activity (mean ± SD, *n* = 10)

After a 1-week rest interval, the animals were then further tested using the open field activity test. Their locomotor activity is shown (Fig. 3a, b). There was no significant change in total movement in the THP group compared with the NS group. All groups travelled similar distances and spent more than half the time in the outer zone and less than 10 % of the time in the center of the apparatus during the 5-min test period.

### THP affects neuroimmune/neuroinflammation genomic transcription patterns after long-term exposure

**Fig. 4:**
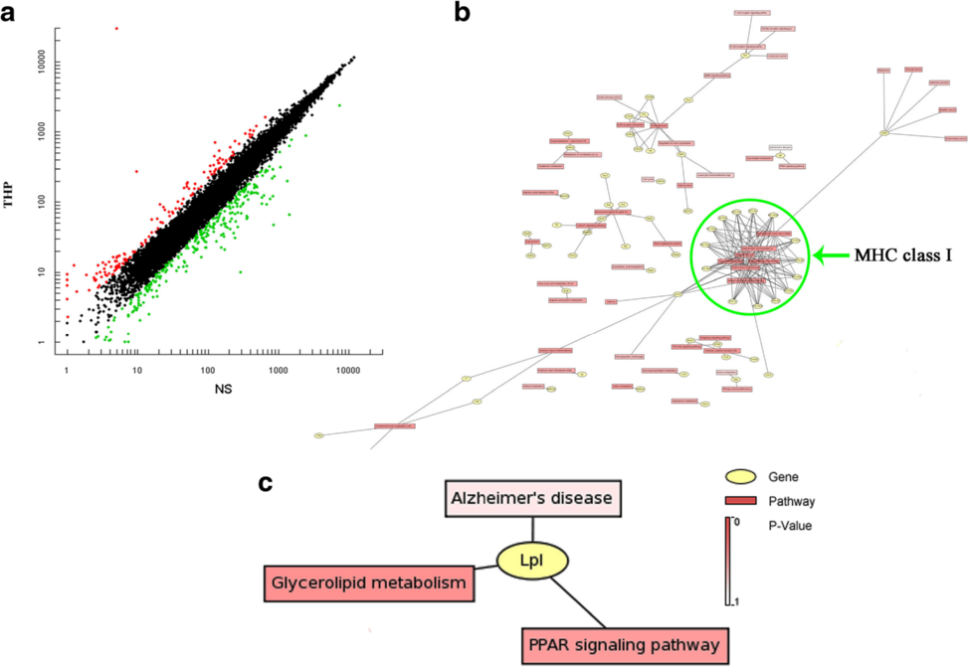
Long-term THP treatment recruits distinct gene expression profiles. **a** Scatterplot of hybridizing signal intensity on DNA microarray. Genetic scatterplot showing differentially regulated hippocampal genes compared with age-matched, NS-treated animals after long-term THP treatment. The *X* and *Y* axes represent fluorescence intensities of Cy3 and Cy5 signals, respectively. Every spot corresponds to the cross-fertilization signal of one gene. *Red* represents upregulation while *green* shows downregulation. **b** Gene transcription profiling reveals functional categories regulated by THP in the hippocampus of aging rats, perculair for MHC class I complex with the highest significance as shown in *green circle area*. And **c** only one common transcript Lpl correlated with Alzheimer’s disease was found and differentially regulated by THP, but less significantly (*P* = 0.5843). These functional pathways were derived from KEGG database analyses

After behavioral testing, hippocampal gene expression profiles were determined by microarray analysis. From a total of 31,043 genes, log-log scatter plots identified 244 differentially modulated genes between THP-treated and age-matched, NS-treated rats (FDR < 0.05 and FC > |2.1|) (Fig. 4a). Of these 244 genes, 68 (47 %) were upregulated, and 176 (53 %) downregulated (the THP-treated group show reduced genetic expression levels compared with the NS group). These differentially expressed genes were subjected to pathway analysis using a MAS functional annotation tool (http://bioinfo.capitalbio.com/mas3) and enriched genes examined by GO and KEGG molecular pathway analyses.

The top ten biological process terms associated with the highest statistical significance by GO pathway analyses are listed in Additional file 1: Table S1. GO pathway analysis of the 176 downregulated genes showed that the most significant affected population was *antigen processing and presentation* (*P* = 2.12E−37). In particular, the GO term indicated by cellular component that showed *MHC class I protein complex* had the highest significance of all 23 genes (Table 2), as well as the KEGG showed by signaling pathway analysis in Fig. 4b. And KEGG signaling pathway analysis revealed that in THP-treated rats, a significant number of pathways were related to graft-versus-host disease, allograft rejection, and autoimmune thyroid disease as shown in (Additional file 2: Table S2). It was notable that several MHC-related genes (e.g., *RT1-CE12*, *CD74*, *RT1-Aw2*, and *Mr1*) are clearly downregulated because these genes are grouped in the *immune response and antigen processing and presentation* and *neuroimmune/neuroinflammatory functions* functional clusters (Additional file 1: Table S1; Additional file 2: Table S2). Both are crucial components of antigen processing and presentation and were significantly different between THP- and NS-treated, age-matched rats. Other highly statistically significant differences among the regulated genes were found for *neurotransmitter transport* and *neuroactive ligand-receptor interaction* (Additional file 1: Table S1; Additional file 2: Table S2), including *Oxt*, *Adra1b*, *Tac1*, *Mas1*, and *Nts*. These were grouped together under *neuroactive ligand-receptor interaction* and *neurological system process* functional clusters (*P* = 9.55E−05). Overall, the neurological system processing and neuroimmune/neuroinflammatory dysfunction appears to be associated with pathological processes induced by THP. Unexpectedly, we noted that only one common transcript, *Lpl* (Fig. 4c), correlated with Alzheimer’s disease and was differentially regulated, albeit less significantly (*P* = 0.5843), using KEGG pathway analysis.

**Table 2:** List of the top five cellular components related to different gene expression between the THP-treated and NS-treated aging rat index by the GO cellular component term

GO term	Count	Protein	*p* value	*q* value
MHCI complex (MHCI)	23	RT1-Aw2; RT1-149; ENSRNOP00000047071; Q6MG05_RAT; RT1-CE10; ENSRNOP00000057803; Q6MG34_RAT; Q861Q3_RAT; ENSRNOP00000041900; Q6MGB8_RAT; ENSRNOP00000047152; Q6MGB9_RAT; Q6MG28_RAT; RT1-Cl; RT1-CE14; Q9JHM2_RAT; RT1-Aw2; XP_001070321; Q6MG29_RAT; Rt1.aa; XP_001072758; Q6MG32_RAT; Q861Q4_RAT; XP_001052972	1.04E−37	1.28E−35
Extracellular region	40	Mgp; Igf2; Anxa2; Bgn; Lgals1; Igfbp2; Fmod; Lum; Ptgds; Penk; Dpp4; Oxt; Gdf10; Cartpt; Tac1; Col1a2; Fn1; DEF3B_RAT; Q6MG90_RAT; C4; Col3a1; Nid1; Rbp4; Serping1; Aebp1; ENSRNOP00000046622; Frzb; Cbln1; Kazald1; Klk8; Omd; Avp; Col1a2; Cklf; Cklf; Bmp6; Ccl6; Bmp7; Timp3; Cxcl13; Nts; Chrdl1	1.22E−33	5.09E−32
Extracellular space	20	Lgals1; Ptgds; Lcat; Oxt; Cp; Cp; Cp; Gdf10; Cartpt; Ada; Col1a2; DEF3B_RAT; Q6MG90_RAT; C4; Rbp4; Serping1; Klk8; Lpl; Col1a2; Cklf; Cklf; Bmp6; Ccl6; Bmp7	1.15E−23	2.81E−22
Myosin	14	Myh11; ENSRNOP00000047484; XP_001053321; XP_001053402; XP_001053536; XP_001073030; XP_001072354; XP_001078110; XP_001076239; XP_576790; XP_001072168; XP_576882; XP_001065390	6.34E−21	1.12E−19
Membrane	51	Ptgds; Slc13a3; Dab2; Enpep; Thbd; Trpc4; ENSRNOP00000052645; ENSRNOP00000052642; ENSRNOP00000052641; ENSRNOP00000052640; ENSRNOP00000052638; ENSRNOP00000052636; ENSRNOP00000008172; ENSRNOP00000052635; Ada; Pln; RT1-Da; ENSRNOP00000003349; Colec12; XP_001061172; MGC108823; XP_574157; Thbd; Fmo1; Cklf; Cklf; ENSRNOP00000058354; INIB_RAT; RT1-Aw2; RT1-149; ENSRNOP00000047071; Q6MG05_RAT; RT1-CE10; ENSRNOP00000057803; Q6MG34_RAT; Q861Q3_RAT; ENSRNOP00000041900; Q6MGB8_RAT; ENSRNOP00000047152; Q6MGB9_RAT; Q6MG28_RAT; RT1-Cl; RT1-CE14; Q9JHM2_RAT; RT1-Aw2; XP_001070321; Q6MG29_RAT; Rt1.aa; XP_001072758; Q6MG32_RAT; Q861Q4_RAT; XP_001052972; Clic2; ENSRNOP00000001372	2.89E−14	3.59E−13

**Fig. 5:**
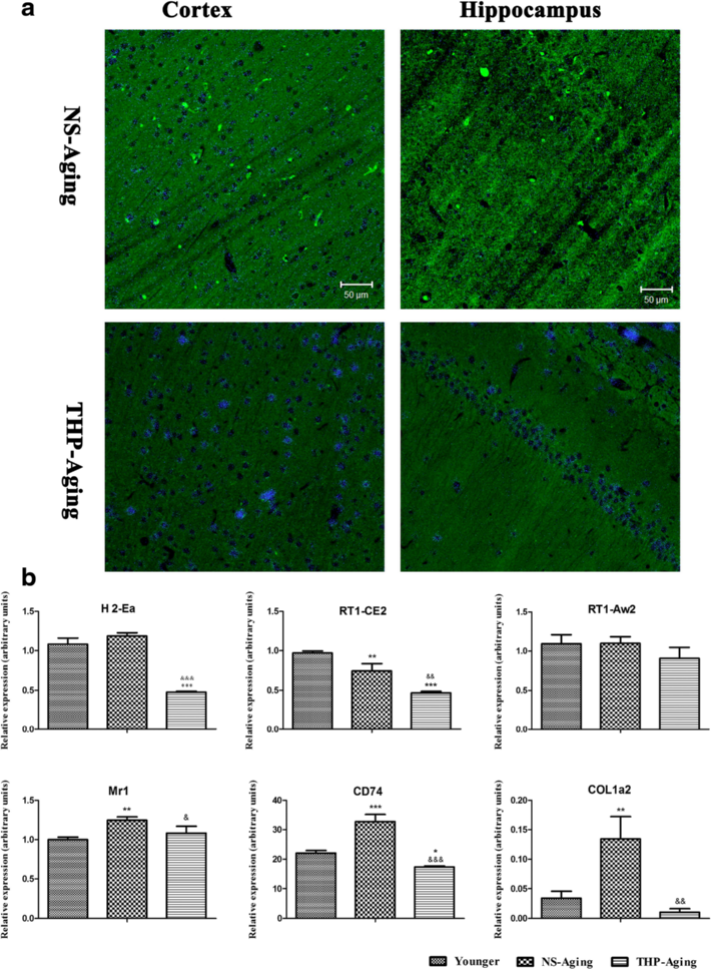
Alterations in MHC-related genes and MHCI protein induced by long-term THP treatment. **a** Confocal analysis of THP-treated aging brains revealed decreased MHCI-positive cells. Scale bar = 50 μm. **b** qRT-PCR revealed mRNA encoding MHC genes (*H2-Ea*, *MR1*, *Cd74*, *RT1-CE12*), and *COL1a2* is significantly downregulated by THP in the hippocampus (mean ± SD, *n* = 3). *P < 0.05, **P < 0.01 compared with the younger group (3 months old); &*P* < 0.05, &&*P* < 0.05 compared with the NS-treated aging group (Dunnett’s test)

To confirm our gene expression profiling data obtained by microarray, we performed qRT-PCR using six selected MHC-relative genes (*H2-Ea*, *Mr1*, *Cd74*, *RT1-CE12*, *COL1a2*, and *RT1-AW2*). Gene expression levels of *RT1-AW2*, *H2-Ea*, *MR1*, *Cd74*, *RT1-CE12*, and *COL1a2* were significantly decreased compared with NS-treated, age-matched rats (Fig. 5b; &*P* < 0.05, &&*P* < 0.01, &&&*P* < 0.001), thereby corroborating our microarray results. Furthermore, expression of MHCI protein in the hippocampus and cortex was investigated by immunofluorescence confocal analysis. Compared with age-matched, NS-treated rats, the number of MHCI-positive cells was significantly decreased in the hippocampus and cortex after THP treatment (Fig. 5a).

### Tau neurodegeneration appears in the brain of rats with long-term THP exposure

**Fig. 6:**
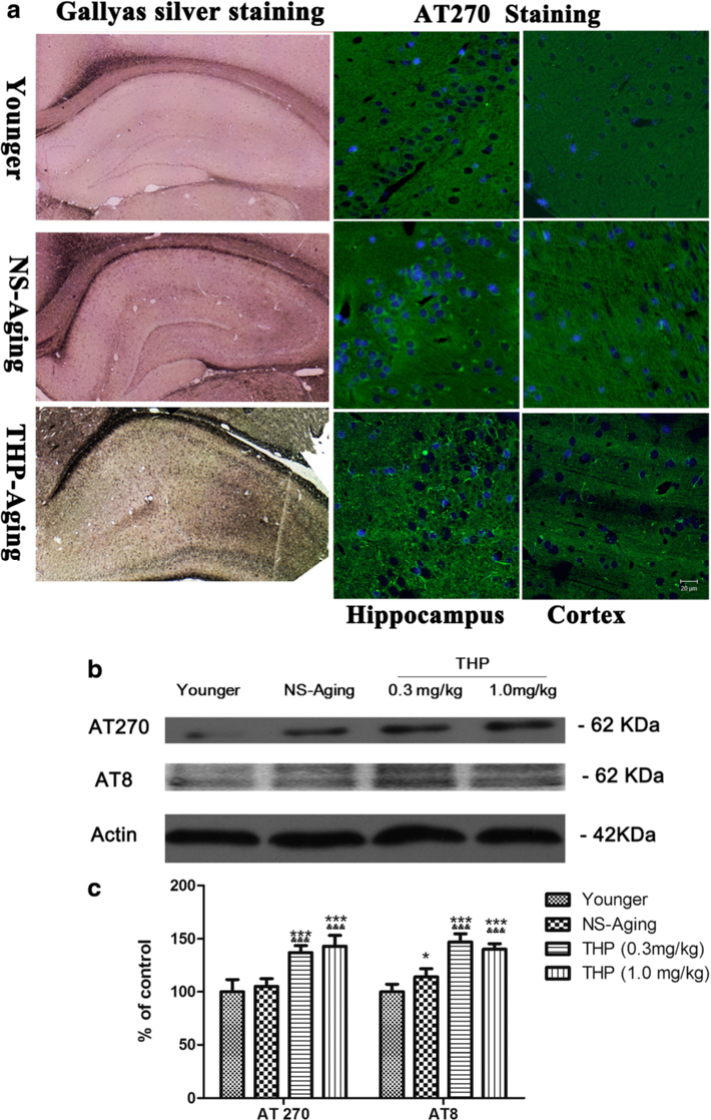
Qualitative and quantitative profiles of neurofibrillary pathology in rats after long-term THP treatment. **a** Neurofibrillary tangles stained by *Gallyas* silver staining in the hippocampus and immunolabeling with antibody AT270 recognizing tau phosphorylated at Thr181. *Gallyas* silver staining revealed more silver-positive staining with a enhanced expression profile of AT270-positive neurofibrillary tangles in the brain of rats with long-term THP exposure. **b** Neurofibrillary pathology was quantified by western blot analysis in the prefrontal cortex as a brain region of interest. **c** Qualitative analysis revealed significantly upregulated AT8 and AT270 expression in THP-treated aging rats compared with the NS-aging group. *P < 0.05, ***P < 0.0001 compared with the younger group (3 months old); &*P* < 0.05, &&&*P* < 0.0001 compared with the NS-treated group (Dunnett’s test). Scale bars = 20 μm

Neurofibrillary pathology was detected in the brain by *Gallyas* silver staining and immunodetection with monoclonal anti-AT270 antibody, which recognizes tau protein phosphorylated at Thr181 (Fig. 6a). In younger rats (i.e., 3-month-old rats), scanty AT270-positive immunoreactivity and less silver-positive neurofibrillary tangles were detected in the somatodendritic compartment of the hippocampal neurons. Compared with age-matched, NS-treated rats, more silver-positive staining with a significantly higher number of AT270-positive neurofibrillary tangles was detected in the brain of rats following long-term THP exposure (Fig. 6a). Similarly, Western blot analysis revealed a significant increase in AT8 and AT270 immunoreactivity in the brain of THP-treated rats (*F* = 70.63, *P* < 0.0001) (Fig. 6b, c).

**Fig. 7:**
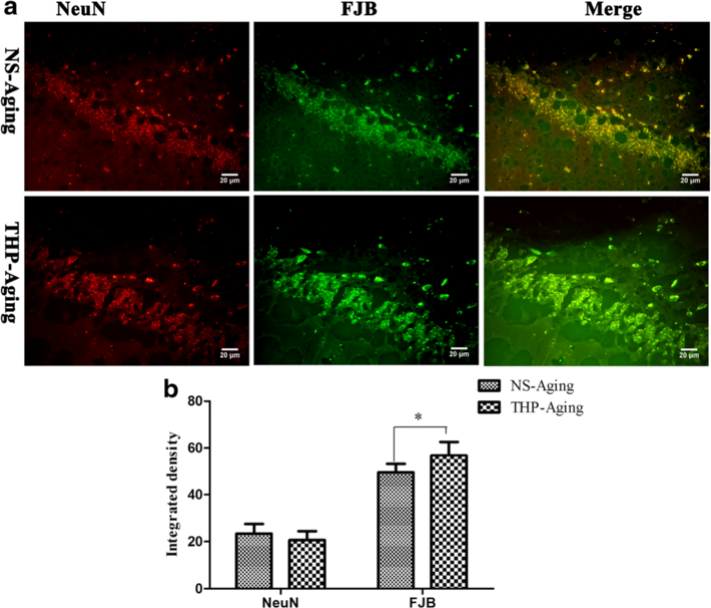
Confocal microscopy revealed hippocampal neurodegeneration in rats after long-term THP treatment. Hippocampal neuronal loss and neurodegeneration were detected using NeuN and FJB, fluorescent markers for neurons and neurodegeneration, respectively. **a** Confocal microscopy shows colocalization of some neurons with neurodegeneration. Scale bar = 50 μm. **b** Integrated immunofluorescence density analysis was performed using ImageJ. THP-treated rats show slightly increased FJB-labeled cells in selected hippocampal areas. ^*^ *P* < 0.05 compared with the NS-treated aging controls (Student’s *t* test)

To determine if the hippocampal neurodegeneration is induced following long-term THP exposure, we used FJB as a fluorescent marker for neurodegeneration, in combination with neuronal marker NeuN staining. Double-labeling studies showed an overlapping regional distribution of NeuN and FJB immunoreactivity in the hippocampus (Fig. 7a). Compared with age-matched, NS-treated rats, THP-treated rats exhibited slightly increased FJB-labeled cells in selected brain section areas (*F* = 2.537, *P* < 0.05) (Fig. 7b), showing that long-term THP exposure induces neurodegeneration in aging rats.

### Long-term THP exposure affects neuroinflammatory patterns in rats

**Fig. 8:**
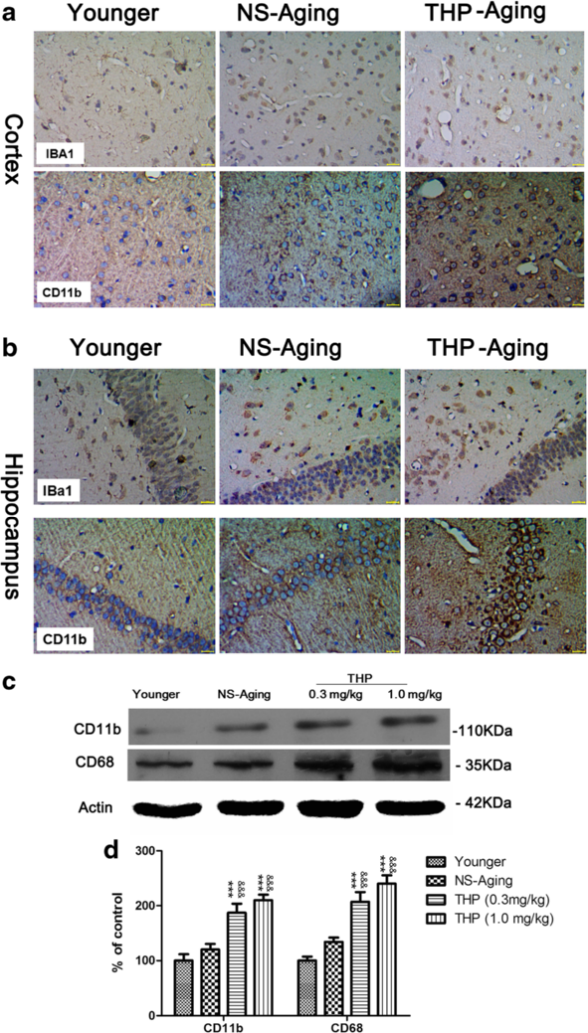
Qualitative profile of activated microglia in the rat brain after long-term THP treatment. **a** and **b** show IBa1 and CD11b expression in the cortex and hippocampus, respectively. In animals following long-term THP treatment, microglia increased in number (IBa1) and became activated (CD11b), with altered morphology and upregulated CD11b. Scale bar = 50 μm. **c**, **d** Activated microglia were quantified by Western blot analysis of cortical CD11b and CD68 expression. &*P* < 0.05, &&&*P* < 0.0001 compared with the NS-treated group (Dunnett’s test)

**Fig. 9:**
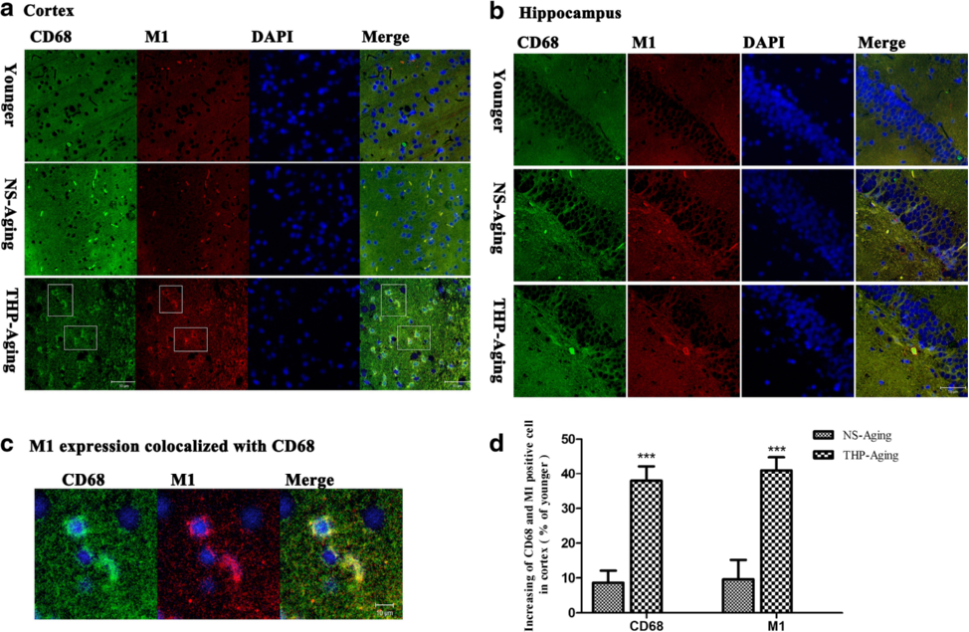
Increased M1 muscarinic receptor expression on activated microglia in the rat brain after long-term THP treatment. Portions of round-shaped cells are positive for CD68 (lysosomal membrane glycoprotein), indicating putative phagocytosis (**a**–**c**). The number of CD68 or M1-positive cells was stereologically quantified in the prefrontal cortex as a region of interest (**d**). Compared with NS-treated aging controls, stereological analysis revealed a significantly higher number of CD68 cells in THP-treated rats. There is also a tendency of increased M1 expression co-localized with CD68 on activated microglia in the rat cortex after long-term THP treatment (**c**, **d**). Scale bar = 50 μm (**a**, **b**); scale bar = 10 μm (**c**). ***P < 0.0001, compared with the NS-treated aging controls (Student’s *t* test)

In the brain, extensive inflammation mediated by reactive microglia was observed using antibodies against IBa1 and CD11b to label microgliosis and activated microglia, respectively. In THP-treated rats, baseline level activity of microglia was minimal and hardly visible in younger rat brain after IBa-1 and CD11b immunocytochemical staining. IBa-1 and CD11b labeling of microglia was enhanced in the hippocampus and cortex of NS-treated, aging rats (Fig. 8a, b). Moreover, we found that this enhanced microglia response positively correlated with the rat’s THP-treated brain, with all dosed groups showing increased microglial numbers and morphological evidence of activation (thick shortened processes, round-shaped cell parts positive for CD68 (lysosomal membrane glycoprotein) indicate putative phagocytosis and cell cluster formation) (Fig. 9a, c). Long-term THP treatment increased microglial expression of the activation marker, CD11b and CD68, in the cortex (Figs. 8a and 9a) and hippocampus (Figs. 8b and 9b). At the same time, immunoblotting revealed significant upregulation of CD11b and CD68 levels in THP-treated aging rats (Fig. 8c, d). Thus, these data suggest that THP-primed hippocampal and cortical microglia undergo an inflammatory phenotypic switch. Two-way ANOVA found that the THP-treated factor creates approximately 92.04 % of variability in microglial activation (*F* = 214.28, *P* < 0.001) compared with control rats.

Notably, our immunofluorescence assays revealed significant upregulation of M1 muscarinic receptor expression co-localized with CD68, which was mainly distributed on microglia/macrophages in THP-treated aging rats (Fig. 9a–c). Stereological quantification revealed the same tendency, with upregulated M1 (*t* = 10.37, *P* < 0.0001) and CD68 (*t* = 12.12, *P* < 0.0001) in the brain cortex of THP-treated aging rats compared with the age-matched NS group (Fig. 9d). These results indicate direct modulation of THP on activated microglia-linked M1 receptors.

## Discussion

Experimental and clinical studies consistently show that cholinergic system dysfunction has a detrimental impact on cognitive performance. Clinical studies suggest that AC agents may produce cognitive side effects among older adults, and duration of AC administration has been described as a risk factor for the appearance of dementia in PD patients. The putative mechanisms may be associated with dysregulation of cholinergic neurotransmission, thereby leading to memory impairments. Nevertheless, the exact mechanisms remain unclear. Here, we report novel findings that long-term THP administration disrupts neuroimmune signaling, with transcriptional activity of antigen processing and presentation noticeably downregulated in the hippocampus of rats. Additionally, we found exacerbated neuroinflammation with misfolded tau protein pathology related to neurodegeneration.

Aging is a multifactorial process that leads to altered behavioral function, including learning and memory. Here, we assessed AC treatment duration as a risk factor for cognitive performance, with THP administration for more than 7 months in aging rats. Thus, we assessed behavioral performance of NS-aging and THP-treated, aging rats in the MWM and open field tasks. The MWM is a task that is commonly used to assess rodents’ spatial learning and memory ability, and the normal function of the hippocampus is essential for this task [17, 18]. In the present study, animals were tested in place navigation and probe trials. It should be noted that only rats treated with THP (1.0 mg/kg, IP) displayed a significantly poor performance in spatial tasks during the initial 3-month training session, in which escape latency was significantly disrupted. It is known that THP acts as a muscarinic receptor antagonist, and muscarinic acetylcholine receptors play key roles in facilitating cognitive processes. This impairment is consistent with several studies using nonselective muscarinic receptor antagonists to block cholinergic signaling in aging and AD rodent models [19, 20]. However, we did not find memory disruption among THP-dosed and NS age-matched groups upon repetition of MWM training following subsequent treatments, with escape latency or swimming speed showing no significant impairment. Moreover, after the final place navigation training, all tested groups generated similar path length percentages during the probe task, while the THP (0.5 mg/kg) group spent significantly more time in the target quadrant of the apparatus compared with the other two groups. The probe data confirm that memory disruption is only present early on in THP treatment. In addition, after the MWM test, THP- and NS-treated rats showed no significant behavioral discrepancies in the open field test. THP and the behavioral deficit in memory retrieval at the start of place navigation may reflect its acute function in blocking muscarinic receptors.

Using microarray analysis, the global impact of THP on the hippocampal gene expression was examined. GO and KEGG pathway analysis showed that the most significantly affected GO category for long-term THP treatment was *immune response and antigen processing and presentation* or *neuroimmune/neuroinflammatory functions*. In particular, genes related to the function of the MHC class I protein complex were significantly downregulated (Table 2 and Fig. 4b). These data were verified by qRT-PCR and confocal-immunofluorescence analyses, with MHC-related genes and MHCI expression significantly downregulated in the rat brain (Fig. 5a, b). Hence, our microarray findings show that long-term THP treatment suppresses innate and adaptive immune homeostasis in laboratory animals. This indicates that THP may function either directly or indirectly in several immune responses and neuroinflammation.

Next, we found that subchronic THP exposure could prime pronounced neuroimmune/neuroinflammatory dysfunction and neuronal phenotypic changes. Neuroinflammatory abnormalities represent a key difference between THP- and NS-treated, age-matched rats. Specifically, THP-treated rats display significantly more Iba1- and CD11b-positive cells of phagocytic morphology compared with age-matched NS-treated littermates. Immunoblotting confirmed the increase in CD11b- and CD68-positive cells associated with long-term systemic THP challenge.

Notably, our current study shows that in activated microglia, THP specifically upregulates M1 muscarinic receptor expression, with a concomitant increase in CD68 (Fig. 9c). THP is widely used as a nonselective muscarinic antagonist and inhibits acetylcholine transmission via blockage of mAChRs [7, 8]. Our current findings suggest that long-term THP use may upregulate muscarinic M1 receptor expression on microglia, and thereby play a role in stimulating phenotypic changes in microglia. Although we cannot exclude the involvement of other muscarinic receptor types in the observed THP effects on microglia, our results support our previous links between cholinergic modulation and the immune system and inflammation.

Involvement of inflammation is considered to be an important factor in resistance variability or susceptibility to AD [21, 22]. Here, we found different alterations in tau pathology spreading in an AD-like manner. In normal young rat brain (3 months old), there was less tau hyperphosphorylation and neurofibrillary tangles. Notably, rats with subchronic THP exposure showed vulnerability to neurofibrillary degeneration with misfolded tau protein aggregation compared with NS-treated, age-matched rats. Stereological quantification and immunoblotting revealed higher levels of phosphorylated tau AT8 and AT270 changes in THP-treated rats compared with age-matched controls. Long-term THP also accelerated a mature neurofibrillary pathology and neurodegeneration in neurons, and further, confirmed by Gallyas silver and FJB staining in the hippocampus of rat brain. Our study supports the hypothesis that long-term THP exposure affects the progression of neurodegeneration in these aging animals.

Despite the evidence described above, neurofibrillary degeneration coupled with an inflammatory response is not consistent with the cognitive behavioral performance. Our study shows that cognitive performance is disrupted as an acute impairment and is only present early on in THP treatment, likely as a consequence of cholinergic signaling blockage because of AC activity. Notably, this behavioral deficit did not continue or exacerbate upon repeated behavioral tests with subsequent THP treatment. Indeed, performances in the MWM and open field tests suggest that chronic THP exposure could lead to adaptation alteration or may be a compensatory mechanism to support cognitive performance, even with loss of neuronal activity. Accordingly, our study supports the hypothesis that THP impacts on the pathogenesis of neurodegeneration in laboratory animals. However, the neuropathological alterations related to these adaptations have yet to be confirmed. Additionally, our current study indicated THP-induced neuroimmune/neuroinflammatory dysfunction may be an event leading to earlier AD development. The neurodegenerative neurofibrillary pathogenesis that results from THP administration might be an earlier event according to the performance in behavioral tests. Moreover, there has been no systematic confirmation that acute or chronic prescription of such medications leads to transient or permanent adverse cognitive outcomes. Several gaps remain in the existing literature on the previously described association between AC use and clinical cognitive function. The mechanisms of action of medications determined in previous studies are inconsistent, making generation of a comprehensive, clinically useful list of AC medications impractical based on those data sets [23, 24]. Thus, understanding such processes is vital because most drug therapies involve chronic AC administration. Our current study is the first to comprehensively measure the cognitive impact of long-term AC exposure on AD in aged laboratory animals. Neuroimmune/neuroinflammatory dysfunction is presented as one of the key differences between THP- and age-matched, NS-treated aging rats, accompanying deleterious neurodegenerative progression. AD is the most common form of dementia in the older population and is characterized by progressive neurodegeneration of the CNS. While the precise etiology of this disease still remains unknown, it is believed that intracellular accumulation of hyperphosphorylated tau (which forms neurofibrillary tangles) and deposition of extracellular filaments play a critical role in neurodegeneration. Accordingly, tau pathologies are evident in AD [21, 25, 26]. From a molecular perspective, AD is a multifactorial disorder involving the association of genetic and environmental factors. Onset and progression of AD may be influenced by several risk factors such as hypertension, metabolic disorders, and/or inflammatory status [27–29]. Owing to the limitations of our study, we cannot clarify the nature of interactions among THP, CNS inflammatory compartments, and neuronal neurodegeneration in this model. Future studies are necessary to determine how THP induces switching of systemic inflammatory events and progression of neurodegenerative pathology. Furthermore, additional studies examining the mechanism by which THP exerts its effects in more commonly used AD models should be performed. Nonetheless, our current results, together with the literature, provide clear implications for the rational use of ACs in older patients.

## Conclusions

In summary, to our knowledge, this is the first study to clearly show that long-term THP treatment impacts on genes related to immune response pathways, particularly antigen presentation, and propagates neuroimmune/neuroinflammatory dysfunction. Our findings indicate that a potential mechanism of THP relates to its effects on several aspects of the immune response, either directly or via neuroimmune pathways. These effects may play a role in modulating the progression of AD and related neurodegenerative disorders.

## Abbreviations

AC, anticholinergic; AD, Alzheimer’s disease; CNS, central nervous system; FJB, Fluoro-Jade B; Iba1, ionized calcium binding adaptor molecule 1; IHC, immunohistochemical; IP, intraperitoneal; MHC, major histocompatibility complex; MWM, Morris water maze; NS, normal saline; PBS, phosphate-buffered saline; PD, Parkinson’s disease; qRT-PCR, quantitative real-time reverse transcription-polymerase chain reaction; SD, Sprague-Dawley rats; TBT, Tris-buffered saline; THP, trihexyphenidyl

## Additional files

Additional file 1: Table S1. The top ten biological processes related to gene changes between THP-treated and NS-treated aging rat index by GO biological process term. (DOC 40 kb) Additional file 2: Table S2. The top ten signal pathway processes that were differentially regulated according to gene expression changes between the THP-treated and NS-treated aging rat index by KEGG molecular pathway analyses. (DOC 40 kb)
