# Supplementary material for: Long-term trihexyphenidyl exposure alters neuroimmune response and inflammation in aging rat: relevance to age and Alzheimer’s disease
Source: J Neuroinflammation. 2016 Jul 1;13:175. doi: 10.1186/s12974-016-0640-5 (PMC4942910; doi:10.1186/s12974-016-0640-5)
Supplement: Additional file 1: Table S1. — The top ten biological processes related to gene changes between THP-treated and NS-treated aging rat index by GO biological process term. (DOC 40 kb) [file 12974_2016_640_MOESM1_ESM.doc]

Additional file 1:

Table S1. The top ten biological processes related to gene changes between THP-treated and NS-treated Aging rats index by Go biological process term.

| GO Term | Count | Protein | p-Value | q-Value |
| --- | --- | --- | --- | --- |
| Antigen processing and presentation | 24 | RT1-Da;RT1-Aw2;RT1-149;ENSRNOP00000047071;Q6MG05_RAT;RT1-CE10;  ENSRNOP00000057803;Q6MG34_RAT;Q861Q3_RAT;ENSRNOP00000041900;  Q6MGB8_RAT; ENSRNOP00000047152; RT1-CE14;Q9JHM2_RAT;  Q6MGB9_RAT;Q6MG28_RAT;RT1-Cl; XP_001070321;Q6MG29_RAT;  Rt1.aa;XP_001072758;Q6MG32_RAT;Q861Q4_RAT;XP_001052972 | 2.12E-37 | 1.23E-35 |
| Immune response | 26 | Colec12;RT1-Aw2;RT1-149;ENSRNOP00000047071;Q6MG05_RAT;RT1-CE10;  ENSRNOP00000057803;Q6MG34_RAT;Q861Q3_RAT;ENSRNOP00000041900;  Q6MGB8_RAT;Ccl6;Cxcl13;ENSRNOP00000047152;Q6MGB9_RAT;Q6MG28_RAT;RT1-Cl;RT1-CE14;Q9JHM2_RAT;RT1-Aw2;XP_001070321;Q6MG29_RAT;Rt1.aa;XP_001072758;Q6MG32_RAT;Q861Q4_RAT;XP_001052972; | 1.29E-30 | 4.21E-29 |
| Antigen processing and presentation of peptide antigen via MHC class I | 12 | RT1-Aw2;Mr1;RT1-149;Q861Q3_RAT;Q6MGB8_RAT;Q6MGB9_RAT;Q6MG28_RAT;RT1-Cl;Q9JHM2_RAT;RT1-Aw2;Q6MG29_RAT;Rt1.aa;Q6MG32_RAT;Q861Q4_RAT | 2.68E-23 | 6.21E-22 |
| Neurotransmitter transport | 9 | Slc6a20;ENSRNOP00000052645;ENSRNOP00000052642;ENSRNOP00000052641; ENSRNOP00000052640; ENSRNOP00000052638;ENSRNOP00000052636;  ENSRNOP00000008172;ENSRNOP00000052635 | 3.76E-13 | 3.63E-12 |
| Cell-matrix adhesion | 6 | Vtn;ENSRNOP00000003349;Nid1;Sned1;XP_237415;ENSRNOP00000022290 | 1.16E-09 | 8.94E-09 |
| Retinoic acid metabolism | 3 | Rbp1;Aldh1a2;Crabp2 | 4.13E-07 | 1.92E-06 |
| Collagen fibril organization | 3 | Anxa2;Col1a2;Col1a2;Col1a1 | 9.80E-07 | 4.37E-06 |
| Actomyosin structure organization and biogenesis | 3 | Cnn1;Actc1;Acta2 | 4.52E-06 | 1.59E-05 |
| Muscle thin filament assembly | 2 | Actc1;Acta2 | 6.90E-06 | 2.26E-05 |
| Cardiac myofibril assembly | 2 | Actc1;Acta2 | 1.01 E-05 | 3.19 E-05 |
