# Supplementary material for: Long-term trihexyphenidyl exposure alters neuroimmune response and inflammation in aging rat: relevance to age and Alzheimer’s disease
Source: J Neuroinflammation. 2016 Jul 1;13:175. doi: 10.1186/s12974-016-0640-5 (PMC4942910; doi:10.1186/s12974-016-0640-5)
Supplement: Additional file 2: Table S2. — The top ten signal pathway processes that were differentially regulated according to gene expression changes between the THP-treated and NS-treated aging rat index by KEGG molecular pathway analyses. (DOC 40 kb) [file 12974_2016_640_MOESM2_ESM.doc]

Additional file 2:

Table S2. The top ten signal pathway processes that were differentially regulated according to gene expression changes between the THP-treated and NS-treated Aging rats index by KEGG molecular pathway analyses.

| **Pathway** | **Count** | **Gene** | **p-Value** | **q-Value** |
| --- | --- | --- | --- | --- |
| Graft-versus-host disease | 15 | H2-Ea;RT1-Aw2;H2-T24;RT1-T24-1;RT1-CE10;RT1-CE2;RT1-A2;RT1-A3;  RT1-CE14;RT1-Cl;RT1-CE16;RT1-A1;RT1-CE15;RT1-CE12;RT1-CE1 | 2.13E-24 | 2.30E-22 |
| Allograft rejection | 15 | H2-Ea;RT1-Aw2;H2-T24;RT1-T24-1;RT1-CE10;RT1-CE2;RT1-A2;RT1-A3; RT1-CE14;RT1-Cl;RT1-CE16;RT1-A1;RT1-CE15;RT1-CE12;RT1-CE1 | 3.74E-24 | 2.30E-22 |
| Type I diabetes mellitus | 15 | H2-Ea;RT1-Aw2;H2-T24;RT1-T24-1;RT1-CE10;RT1-CE2;RT1-A2;RT1-A3; RT1-CE14;RT1-Cl;RT1-CE16;RT1-A1;RT1-CE15;RT1-CE12;RT1-CE1 | 2.31E-23 | 9.04E-22 |
| Autoimmune thyroid disease | 15 | H2-Ea;RT1-Aw2;H2-T24;RT1-T24-1;RT1-CE10;RT1-CE2;RT1-A2;RT1-A3; RT1-CE14;RT1-Cl;RT1-CE16;RT1-A1;RT1-CE15;RT1-CE12;RT1-CE1 | 2.94E-23 | 9.04E-22 |
| Antigen processing and presentation | 16 | Cd74;H2-Ea;RT1-Aw2;H2-T24;RT1-T24-1;RT1-CE10;RT1-CE2;RT1-A2;  RT1-Aw2;RT1-CE14;RT1-Cl; RT1-A3; RT1-CE16;RT1-A1;  RT1-CE15;RT1-CE12;RT1-CE1 | 2.34E-22 | 5.76E-21 |
| Cell adhesion molecules (CAMs) | 16 | H2-Ea;Cdh1;RT1-Aw2;H2-T24;RT1-T24-1;RT1-CE10;RT1-CE2;RT1-A2;  RT1-CE14;RT1-Cl;RT1-A3;RT1-Aw2;RT1-CE16;RT1-A1;RT1-CE15;  RT1-CE12;RT1-CE1 | 1.74E-19 | 3.57E-18 |
| Natural killer cell mediated cytotoxicity | 14 | RT1-Aw2;H2-T24;RT1-T24-1;RT1-CE10;RT1-CE2;RT1-A2; RT1-A3;  RT1-CE14;RT1-Cl;RT1-CE16;RT1-A1;RT1-CE15;RT1-CE12;RT1-CE1 | 1.07E-16 | 1.89E-15 |
| Focal adhesion | 8 | Vtn;Col1a2;Fn1;Col3a1;Flna;Col1a1;Myl9;Col6a3 | 3.51E-07 | 2.60E-06 |
| Neuroactive ligand-receptor interaction | 7 | Oxt;Adra1b;Tac1;Mas1;Avp;Htr5b;Nts | 9.55E-05 | 4.28E-04 |
| Complement and coagulation cascades | 4 | Thbd;C4b;Serping1;Thbd;C7 | 9.74E-05 | 4.28E-04 |
